# Supplementary material for: Theoretical Studies of Leu-Pro-Arg-Asp-Ala Pentapeptide (LPRDA) Binding to Sortase A of Staphylococcus aureus
Source: Molecules. 2022 Nov 24;27(23):8182. doi: 10.3390/molecules27238182 (PMC9890316; doi:10.3390/molecules27238182)

Supporting information for article “Theoretical studies of Leu-Pro-Arg-Asp-Ala pentapeptide (LPRDA) binding to Sortase A of Staphylococcus aureus”

https://doi.org/10.3390/molecules27238182

Dmitry Shulga, Konstantin Kudryavtsev

Table of Contents

RMSD for MD........1
LPRDA-SrtA MD with 3 replicas for 10 ns........1
Receptor........1
Ligand........3
SrtA (apo form) MD for 10 ns........4
LPRDA-SrtA complex MD for 90 ns (continuation)........4
Receptor........4
Ligand........5
RMSF analysis........7
LPRDA-SrtA complex MD for 90 ns (continuation)........7
Receptor........7

RMSD for MD

LPRDA-SrtA MD with 3 replicas for 10 ns

Receptor

Table S1. RMSD values of the SrtA protein (C-alpha atoms) in LPRDA-SrtA complex.

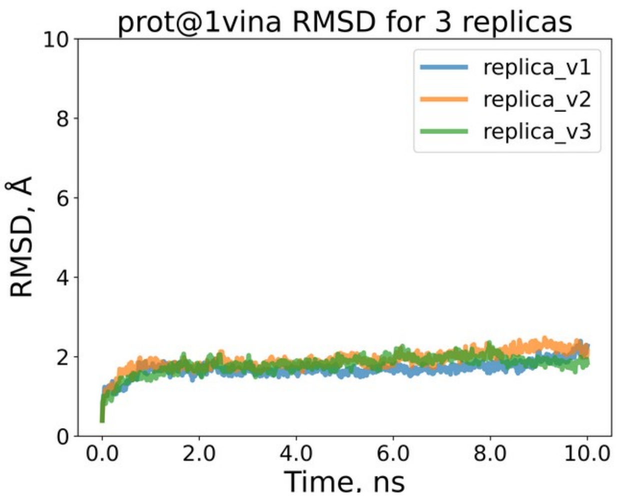 ADV #1, E = -7.3 kcal/mol	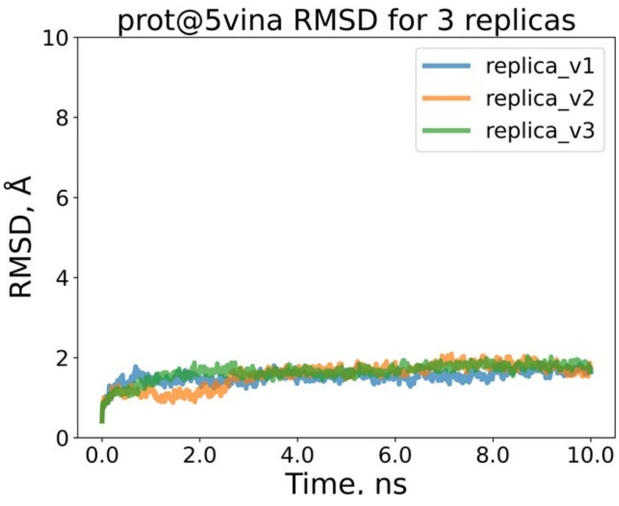 ADV #5, E = -7.0 kcal/mol

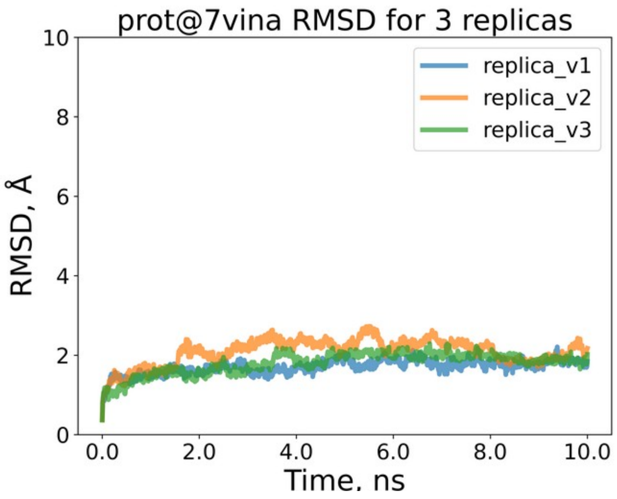

ADV #7, E = -6.9 kcal/mol

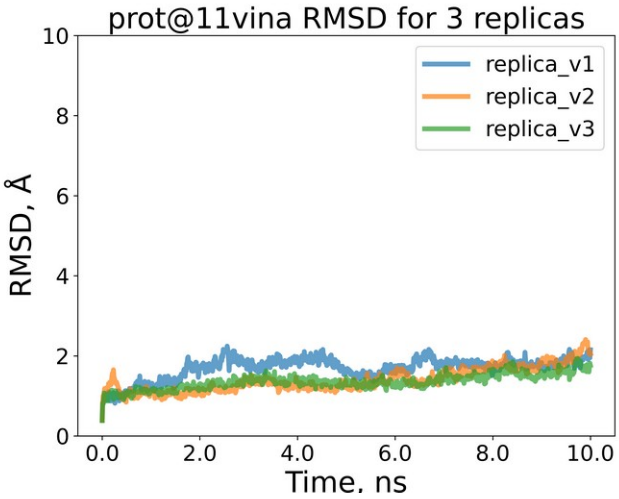

ADV #11, E = -6.8 kcal/mol

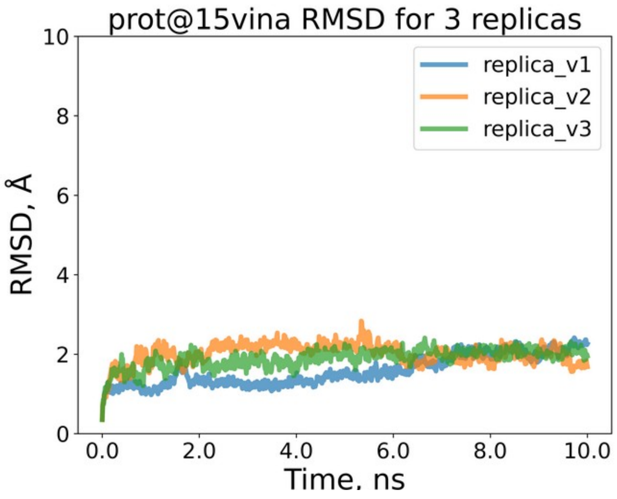

ADV #15, E = -6.6 kcal/mol

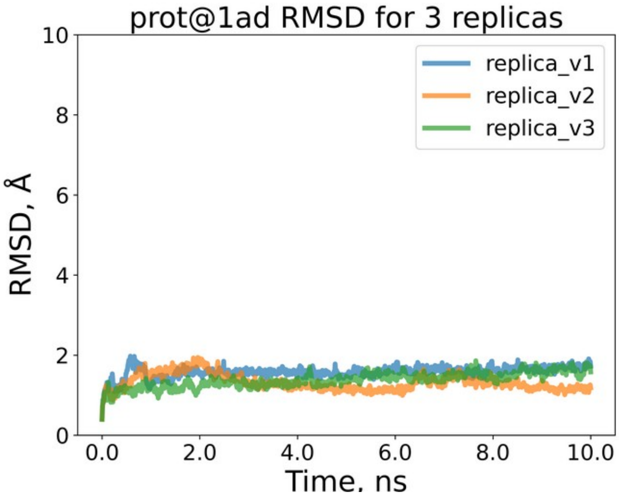

AD #1, E = -7.94 kcal/mol

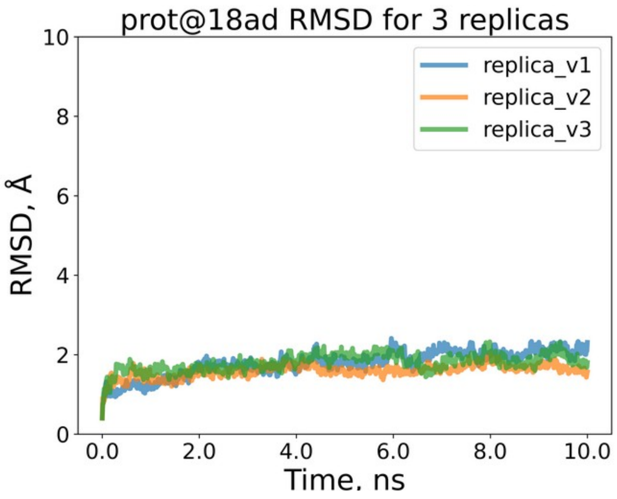

AD #18, E = -5.83 kcal/mol

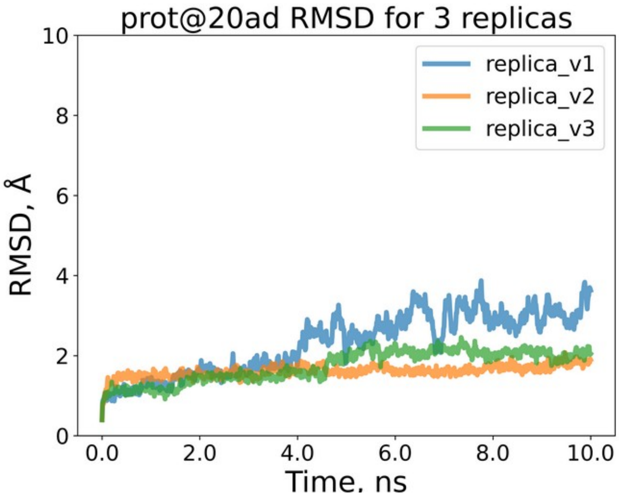

AD #20, E = -5.75 kcal/mol

Ligand

Table S2. RMSD values of LPRDA in LPRDA-SrtA complex.

<table>
<tr>
<td>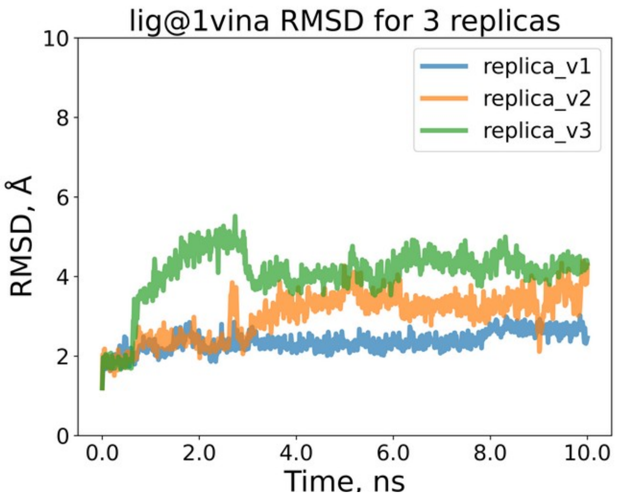

ADV #1, E = -7.3 kcal/mol</td>
<td>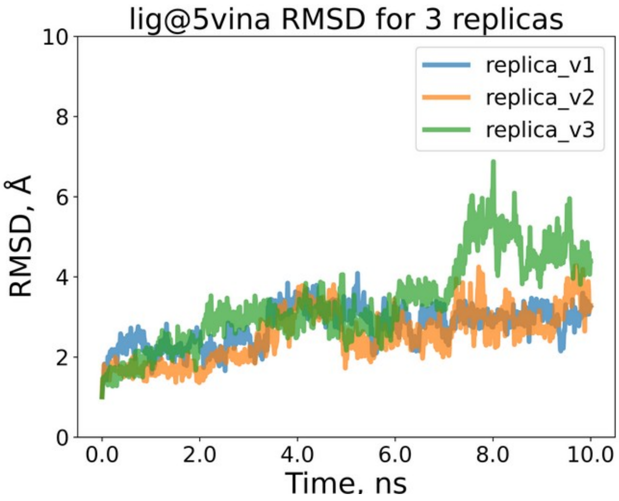

ADV #5, E = -7.0 kcal/mol</td>
</tr>
<tr>
<td>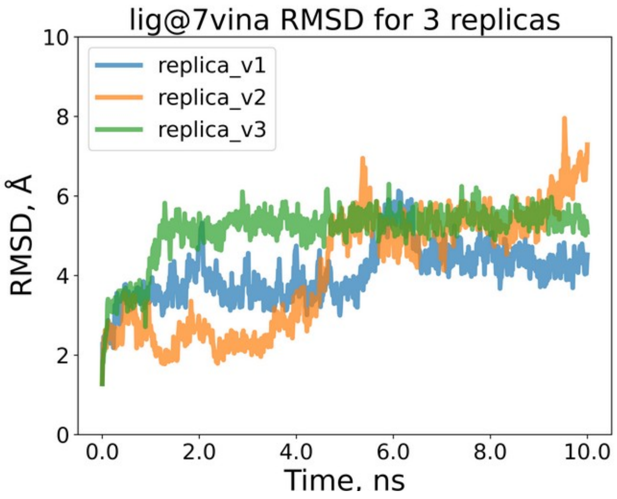

ADV #7, E = -6.9 kcal/mol</td>
<td>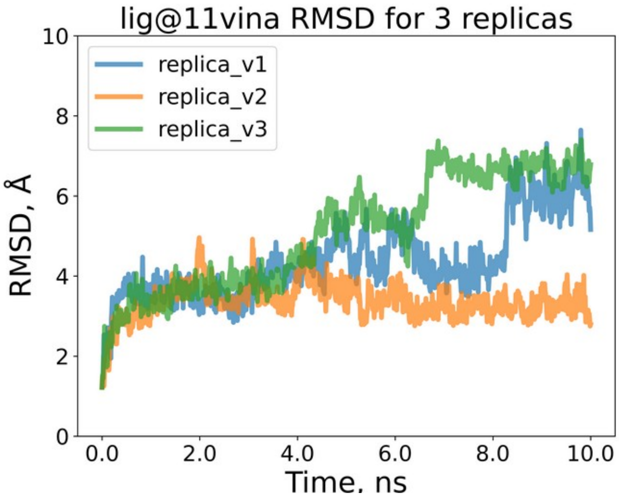

ADV #11, E = -6.8 kcal/mol</td>
</tr>
<tr>
<td>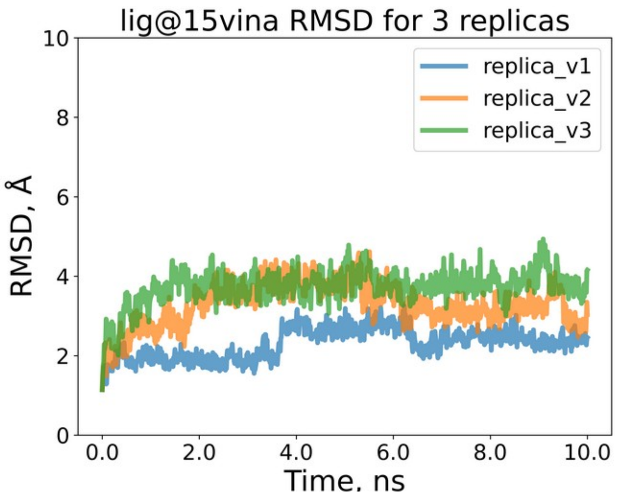
</td>
<td>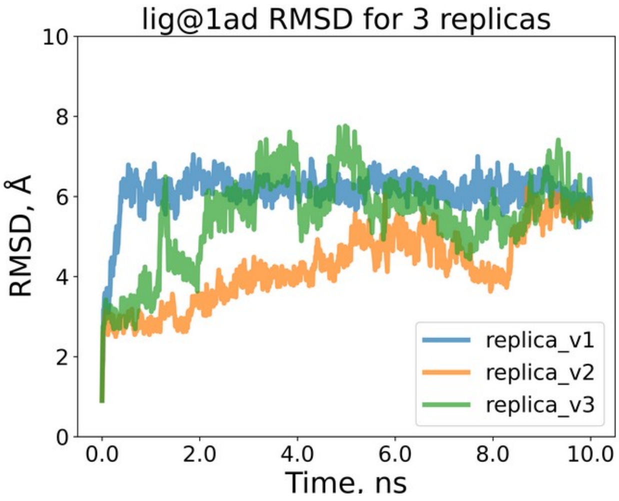
</td>
</tr>
</table>

<table>
<tr><td>ADV #15, E = -6.6 kcal/mol</td><td>AD #1, E = -7.94 kcal/mol</td></tr>
<tr><td>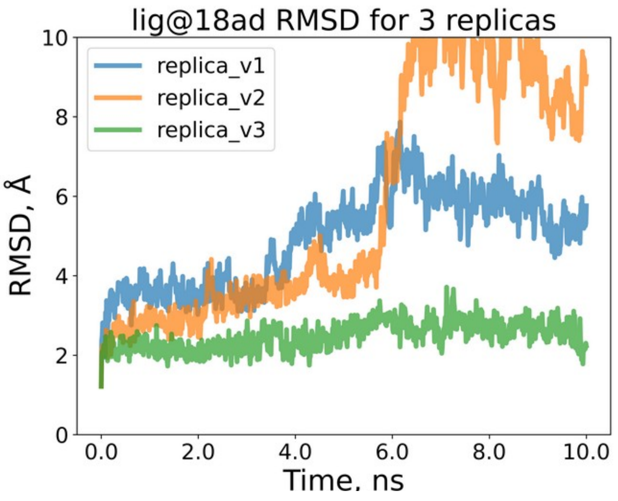

AD #18, E = -5.83 kcal/mol</td><td>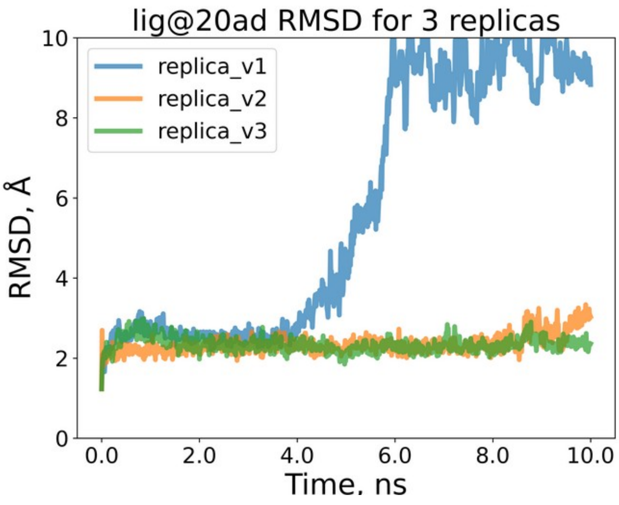

AD #20, E = -5.75 kcal/mol</td></tr>
</table>

SrtA (apo form) MD for 10 ns

Figure S1. RMSD of the apo form of SrtA during the 10 ns MD simulation.

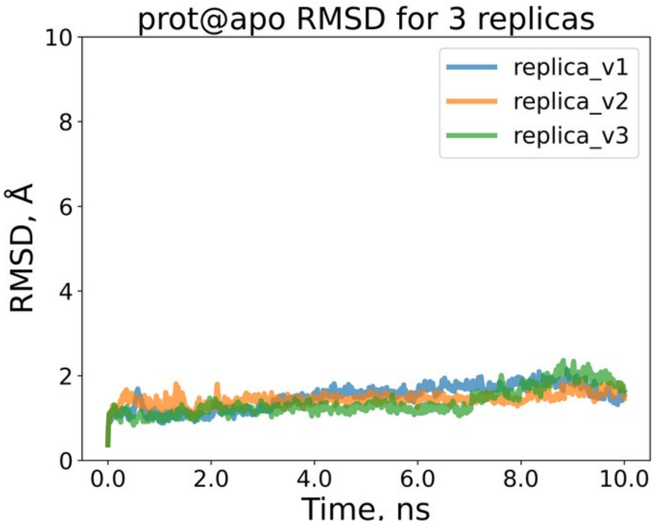

LPRDA-SrtA complex MD for 90 ns (continuation)

Receptor

Table S3. SrtA (C-alpha atoms) RMSD for the continued MD (for another 90 ns) for the selected LPRDA-SrtA complexes. I. ADV #1 (repl.1), II. ADV #7 (repl. 1), III. ADV #7 (repl.3), IV. ADV #15 (repl.1), V. AD #18 (repl.3), VI. AD #20 (repl.3). The starting geometry for each complex was taken from the final geometries of the respective 10 ns MD trajectories.

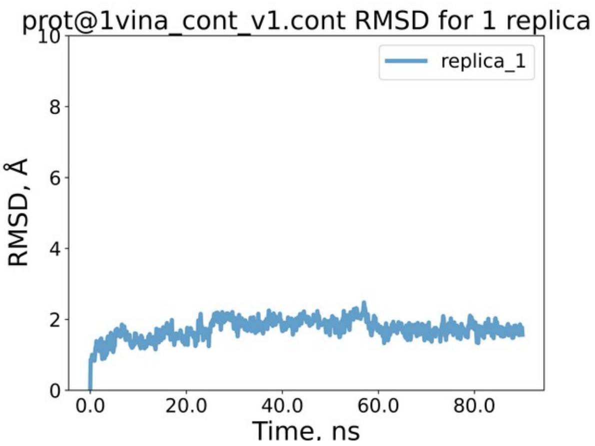

I

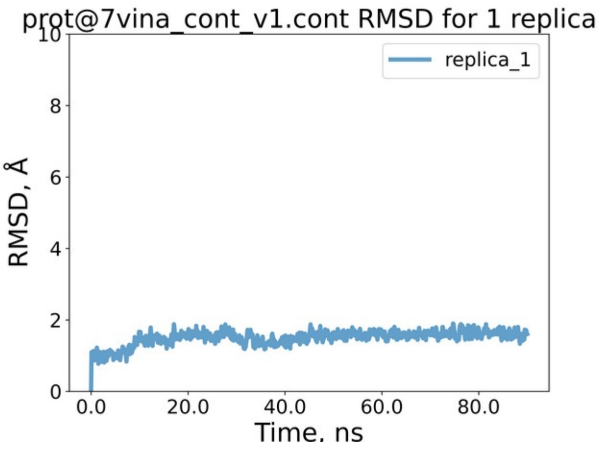

II

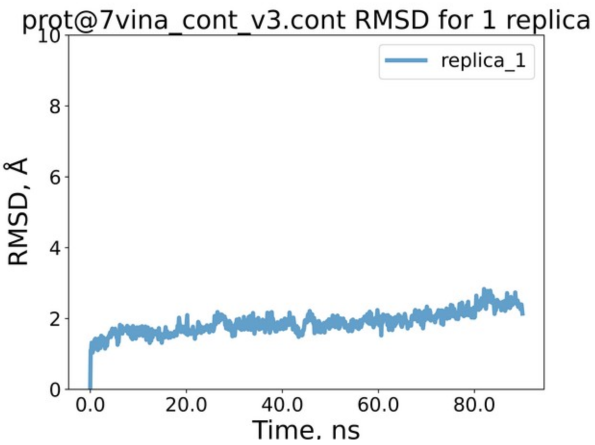

III

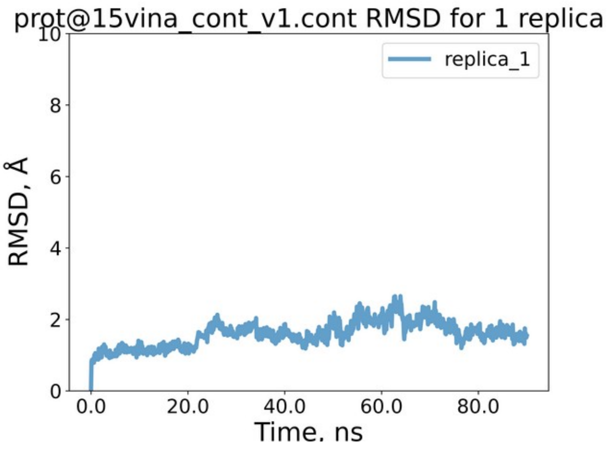

IV

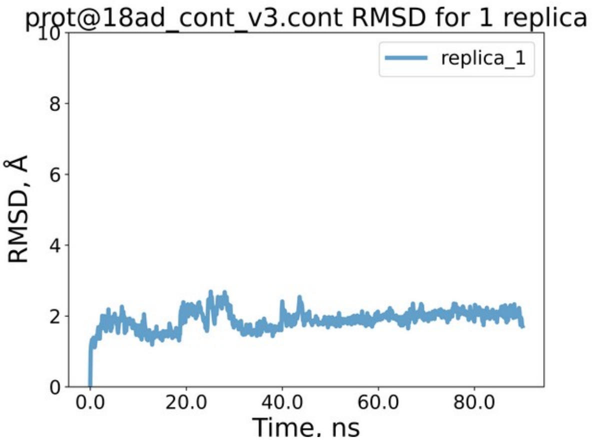

V

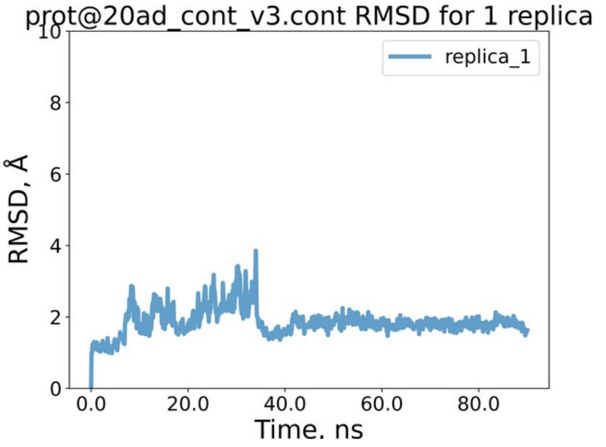

VI

Ligand

Table S4. LPRDA RMSD for the continued MD (for another 90 ns) for the selected LPRDA-SrtA complexes. I. ADV #1 (repl.1), II. ADV #7 (repl. 1), III. ADV #7 (repl.3), IV. ADV #15 (repl.1), V.

AD #18 (repl.3), VI. AD #20 (repl.3). The starting geometry for each complex was taken from the final geometries of the respective 10 ns MD trajectories.

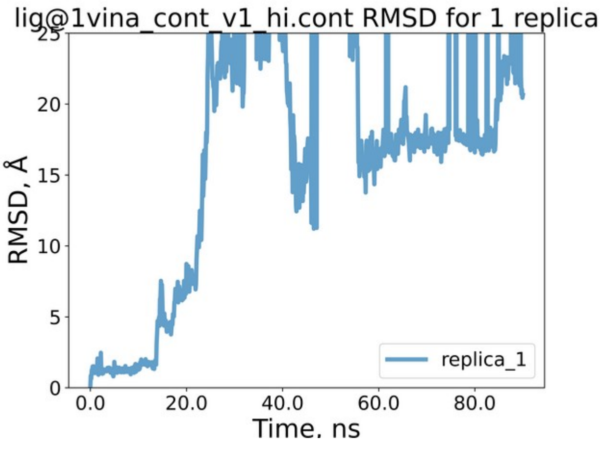

I

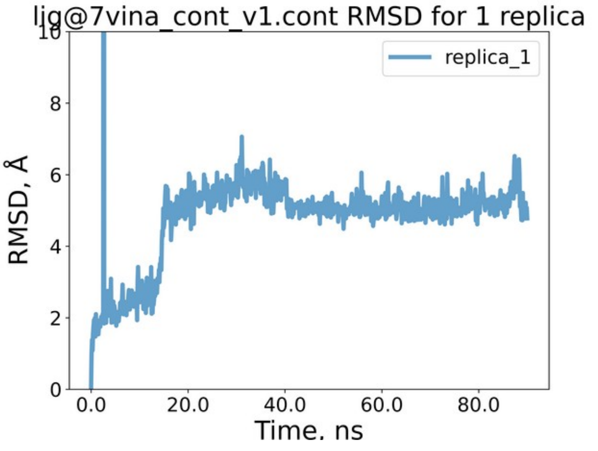

II

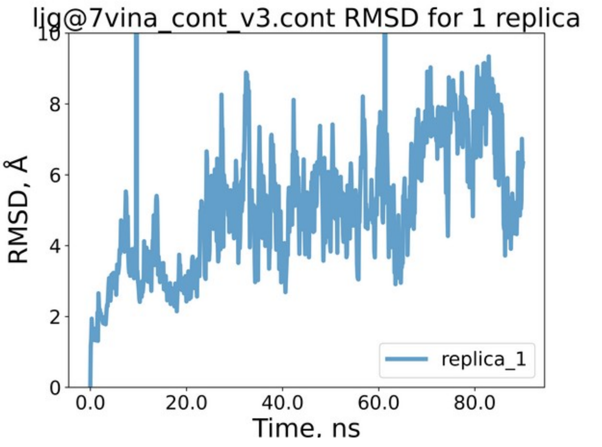

III

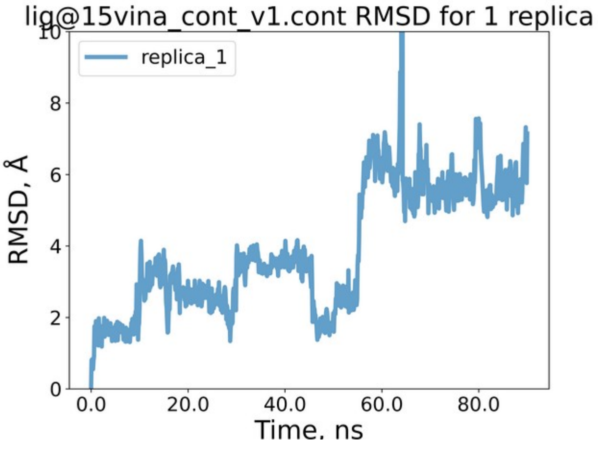

IV

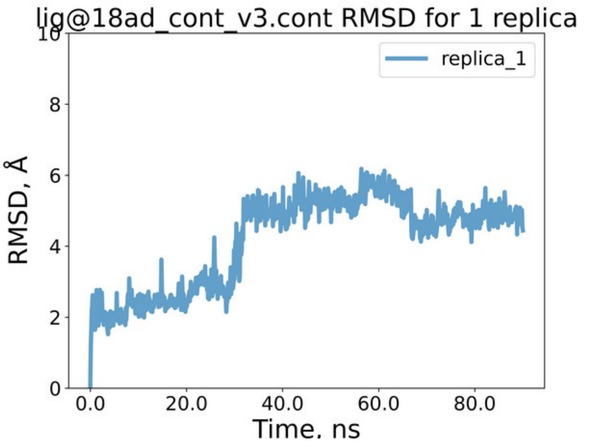

V

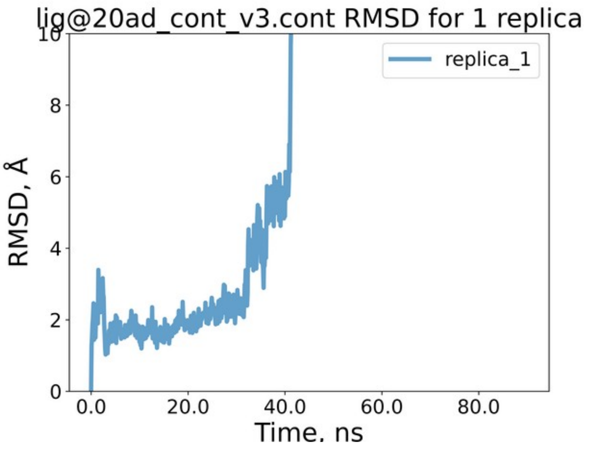

VI

RMSF analysis

LPRDA-SrtA complex MD for 90 ns (continuation)

Receptor

Table S5. RMSF values obtained for the SrtA receptor from 90 ns MD simulation of 6 different LPRDA-SrtA complexes. RMSF values are obtained for C-alpha atoms.

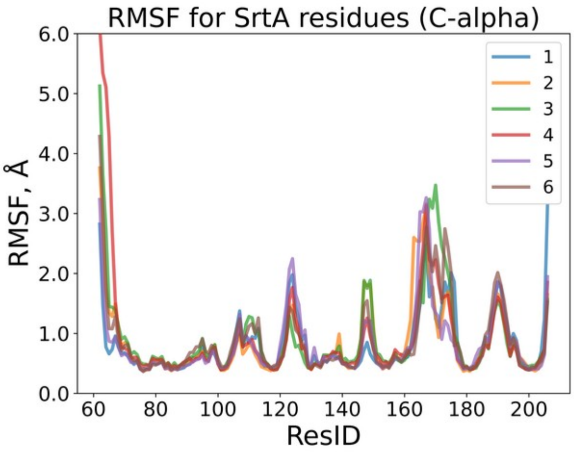

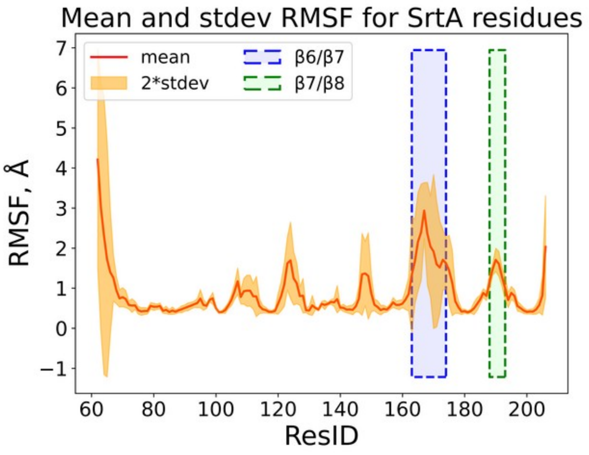

Supplement: Supplementary file 1 [file molecules-27-08182-s001.zip › molecules-2014976-SI.pdf]
